# The influence of antibiotic and mechanical bowel preparation on the microbiome in colorectal cancer surgery: A pilot study

**DOI:** 10.1016/j.sipas.2025.100302

**Published:** 2025-08-06

**Authors:** Undine Gabriele Lange, Konrad Lehr, René Thieme, Albrecht Hoffmeister, Jürgen Feisthammel, Ines Gockel, Alexander Link, Boris Jansen-Winkeln

**Affiliations:** a Clinic and Polyclinic for Visceral, Transplant, Thoracic and Vascular Surgery, Faculty of Medicine, University of Leipzig, Leipzig, Germany; b Department of Gastroenterology, Hepatology and Infectious Diseases, Faculty of Medicine, Otto von Guericke University Magdeburg, Magdeburg, Germany; c Clinic and Polyclinic for Oncology, Gastroenterology, Hepatology and Pneumology, Faculty of Medicine, University of Leipzig, Leipzig, Germany; d Department for General, Thoracic and Emergency Surgery, Hospital Magdeburg, 39130 Magdeburg, Germany; e Clinic for General, Visceral, Thoracic and Vascular Surgery, Clinic St. Georg Leipzig, Leipzig, Germany

**Keywords:** Gut microbiome, Preoperative oral antibiotics, Colorectal surgery, Bowel preparation

## Abstract

**Backround:**

The extent to which bowel preparation mechanical (MBP) or oral antibiotic (OA) or in combination (MBP/OA)) should be performed prior to elective colorectal surgery is the subject of ongoing debate. The aim of our study was to investigate the effect of MPB/OA [with single-shot intravenous antibiotic administration before incision (MPB/OA+ivAB)] on the microbiome of patients with colorectal carcinoma (CRC) operated minimally-invasive.

**Methods:**

We were studying 16 consecutive patients who underwent elective resection surgery for CRC at our centre in a prospective panel study. MBP was performed and 1 g parmomomycin/500 mg metronidazole was administered orally one day preoperatively; 1 g ertapenem was administered intravenously 30 min prior incision. Three mucosal samples were taken preoperatively during colonoscopy and intraoperatively and analysed by 16S rRNA V1-V2 gene sequencing.

**Results:**

Before MPB/OA+ivOA, the genera *Phocaeicola* (10 %), *Bacteroides* (7 %) and *unclassified Ruminococcaceae* (6 %) were predominant. After preparation, all bacteria were reduced except *Enterococcus* (7 %) and *Escherichia/Shigella* (6 %), which had increased. Significant reductions were seen for *Bacteroides* (*p* = 0.01), *Haemophilus* (*p* = 0.047), *Holdemanella* (*p* = 0.004), *Neisseria* (*p* = 0.004), *Odoribacter* (*p* = 0.027), *unclassified Clostridiales* (*p* = 0.008) and *unclassified Ruminococcacaeae* (*p* = 0.009). Large effect sizes (Cohens’d) were seen for *Bacteroidetes* (*d* = 0.864) and *unclassified Ruminococcacaeae* (*d* = 0.909).

**Conclusions:**

In our pilot study, we observed a significant reduction in seven bacterial genera after MBP/OA+ivAB in patients with CRC. Some of these bacterial genera have been associated with anastomotic insufficiency. Further, large in-depth analyses are needed to evaluate perioperative microbial drift with postoperative complications.

## Highlights

• Oral antibiotics reduces bacterial microdiversity in mucosal bowel samples.
• Unlike others, *Enterococcus* and *Escherichia/Shigella* were more prevalent afterwards.
• Effect size was large for reduction of *Bacteroides* and *Ruminococcacaeae*.

## Introduction

Colorectal carcinoma is one of the most frequent tumour entities and therefore the importance of elective colorectal cancer surgery remains significant. But surgical site infections (SSI), especially deep incisional SSI [1], and anastomotic leakage (AL) with life-threating consequences are persistently problematic in this part of the body which has the highest bacterial density with up to 10^12^ bacteria/ml mucosa [2].

As early as 1972, Nichols et al. presented their protocol in which they achieved a reduction in SSI from 43 % to 9 % with the combination of mechanical bowel preparation and oral administration of neomycin/erythromycin [3]. Furthermore, Schardey et al. reported in the early 90 s reduced rates of AL with oral antibiotics [4]. Since then, the combination of mechanical bowel preparation and oral antibiotic administration (MBP/OA) has repeatedly been the subject of clinical studies such as for instance in the prospective randomized SELECT trial [5]. Significant advantages of MBP/OA compared to MPB alone regarding SSI and AL were reported [[6], [7], [8]]. There is evidence that OA alone is sufficient, but further randomized studies are needed. In contrast, the superiority of OA compared to perioperative iv antibiotics (right before incision and if necessary, during surgery) (ivAB) was demonstrated [9]. However, perioperative antibiotic bowel preparation is not yet part of daily practice in European surgical departments and is not yet part of the guidelines [10], as it is the case in America [11].

It has been shown that SSI, AL and postoperative ileus are associated with certain genera in the gut microbiome [[12], [13], [14], [15], [16], [17], [18]]. But the precise effect of preoperative oral antibiotics on the microbiome especially in patients with colorectal cancer has not yet been investigated. Therefore, the aim of our study was to detect changes in the microbiome after MBP/OA+ivAB treatment in mucosal samples in a patient cohort with CRC to create better understanding of OA impact.

## Material and methods

### Study design

The study design is a prospective panel study. 16 consecutive patients who underwent elective minimal-invasive cancer resection with primary anastomosis (with or with colostomy) of the colon or rectum at our centre (University Hospital Leipzig, Saxony, Germany) were recruited. For patient characteristics see Table 1. Three mucosal samples were obtained preoperatively during colonoscopy/rectoscopy (time point: PreSURG; mean 4 [[1], [2], [3], [4], [5], [6], [7], [8], [9], [10], [11], [12], [13], [14], [15]] days before surgery). One day before elective minimal-invasive surgery the patients underwent bowel preparation composed of MBP/OA. MBP included one pack of Moviprep® (contains 5900 mg natrium ascorbate, 2691 mg natrium chloride, 7500 mg natrium sulphate, 1015 mg calium chloride, 100,000 mg Macrogol 3350 dissolved in two litres water). After MBP 1 g paromomycin and 1 g metronidazole were taken by patients orally also a day before surgery. 1 g ertapenem was administered intravenously 30 min prior to incision. The second mucosal tissue collection was intraoperatively (three samples; time point: *SURG*). AL was defined in accordance with an international consensus as a defect in the intestinal wall at the anastomotic site, resulting in a communication between intra- and extraluminal compartments that requires active therapeutic intervention—either without relaparotomy (Grade B) or with relaparotomy (Grade C) [19]. The study was conducted in accordance with the current Good Clinical Practice guidelines and the Declaration of Helsinki and approved by the local ethics committee and government authorities (253/19-ek). All patients gave their written informed consent to the participation in the study and were registered in clinicaltrials.org (NCT03759886). The patients did not receive any probiotic therapy before participating in the study.

**Table 1:** Patients characteristics.

Variable	
Sex, n ( %) male female	12 (75) 4 (25)
Age (years), mean (SD)	60 (±8.01)
Active smoker, n ( %)	3 (18.75)
Diabetes mellitus type II, n ( %)	6 (37.5)
BMI ≥30 kg/m^2^	2 (12.5)
Colon carcinoma, n ( %)	5 (21.25)
Rectal carcinoma, n ( %)	11 (68.75)
(y)UICC stage, n ( %) 0 I II III IV	1 5 6 2 2

Data are presented as absolute number and frequency, continuous data presented as mean ± standard deviation. BMI = body mass index, UICC = Union for International Cancer Control.

### Inclusion and exclusion criteria

Inclusion criteria were: age over 18 years, elective colorectal cancer surgery. Exclusion criteria included preoperatively history of organ transplant, active immunosuppressive medication or chronic steroid use, history of inflammatory bowel disease and use of any antibiotic 30 days before enrolment. Patients with intraoperative tumour perforation should be excluded.

The study was approved by the Institutional Review Board of the University of Leipzig (number 253/19-ek). Informed written consent was obtained from each study participant prior to enrolment.

### Sample collection

A total of 96 mucosal biopsies were collected by biopsy forceps or open surgery at the medical faculty of the University Hospital Leipzig (Saxony, Germany) and placed in 1,8 mL polystyrene containers and immediately transferred to − 80 °C freezer until processing.

### DNA extraction and sequencing

The DNA extraction and sequencing were performed at the medical faculty of the Otto-von-Guericke University in Magdeburg (Saxony-Anhalt, Germany). Samples were suspended in 1 ml of lysis buffer (composed of 100 mM Tris–HCl pH 8.0, 100 mM EDTA, 100 mM NaCl, 1 % (w/v) polyvinylpyrrolidone and 2 % (w/v) sodium dodecyl sulphate) in a 2 mL Lysing Matrix E tube (Qbiogene, Alexis Biochemicals, Carlsbad, USA)). Following, a FastPrep-24 Instrument (MP Biomedicals, Santa Ana, USA) was used for the mechanical lysis (40 s and 6.0 m *s* − 1). The DNA extraction was followed based on phenol/chloroform as previously described [20]. V1-V2 region of the 16S rRNA gene was amplified with 40 cycles PCR reaction and the 27F and 338R primers. The paired-end sequencing was performed on a MiSeq (2 × 250 bp; Illumina, San Diego, USA) [21,22].

### Statistics

After sequencing and demultiplexing all fastQ files were analysed with R Statistical Software version 4.2.1 (2022; R Foundation for Statistical Computing, Vienna, Austria) using the dada2 package version 1.24.0. Microbial community were analysed at the taxonomic rank of family and genus in relative abundances (expressed as percentages, see Suppl. Table 1). The following analyses were conducted using SPSS Statistics version 26 software from International Business Machines Corporation.

For the metric variables considered, there are no indications of an extreme distribution in the sense of floor or ceiling effects. In the absence of a normal distribution (according to the Shapiro-Wilk test) of the sample, the two-sided test is carried out with the Wilcoxon signed-rank test (equal distribution of the groups was tested with the Kolmogorov-Smirnov test). Unless otherwise stated, the test was performed at a significance level corresponding to *p* < 0.05.

## Results

### Patient characteristics

We studied 16 consecutive patients who underwent elective minimal-invasive cancer resection of the colon or rectum. Male patients predominated our cohort (12 male vs 4 female patients). The mean age were 80 years (±8 years). 5 patients underwent elective resection for carcinoma of the colon (three patients for cancer of the right hemicolon and two of the sigmoid). One colon cancer patient suffered from obesity (body mass index (BMI) of 33.4 kg/m^2^). 6 patients have diabetes mellitus type II and 3 patients were active smokers. Two rectal carcinomas were located in the upper third of the rectum (≥12 cm), 8 in the middle (6cm-<12 cm) and one in the lower third (<6 cm). For characteristics see Table 1.

### Predominant bacterial genera during study course

**Fig. 1:**
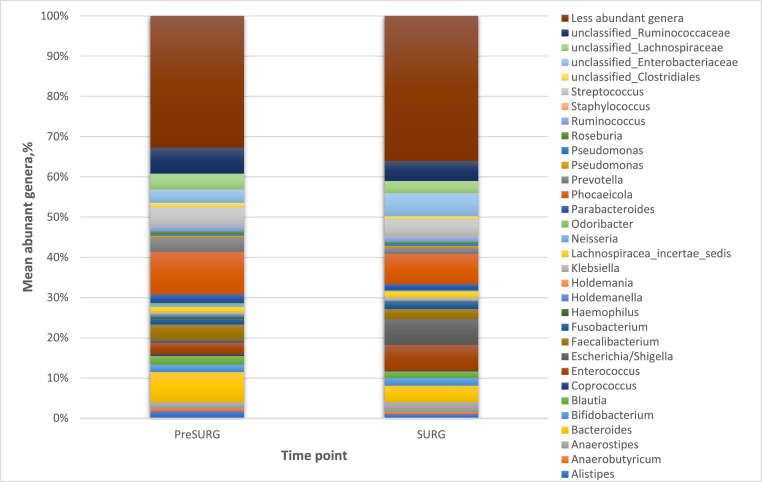
Mean distribution of abundant genera in mucosal samples before and after MBP/OA±ivOA Distribution of abundant genera in patient mucosal samples at the two time-points (PreSURG and SURG).

At the first timepoint (PreSURG) the genera *Phocaeicola* (10 %), *Bacteroides* (7 %) and *unclassified Ruminococcaceae* (6 %) were predominant in the mucosal samples obtained during coloscopy or rectoscopy. After preparation with MBP/OA (single dosis of parmomomycin and metronidazole) and single-shot ertapenem for incision (ivAB) (timepoint: SURG), all bacteria were reduced except of *Enterococcus* (7 %) and *Escherichia/Shigella* (6 %), which had increased (see Fig. 1). The analyses were performed with 16S rRNA V1-V2 gene sequencing.

### Significant changes of genera after MPB/OA±ivOA

**Fig. 2:**
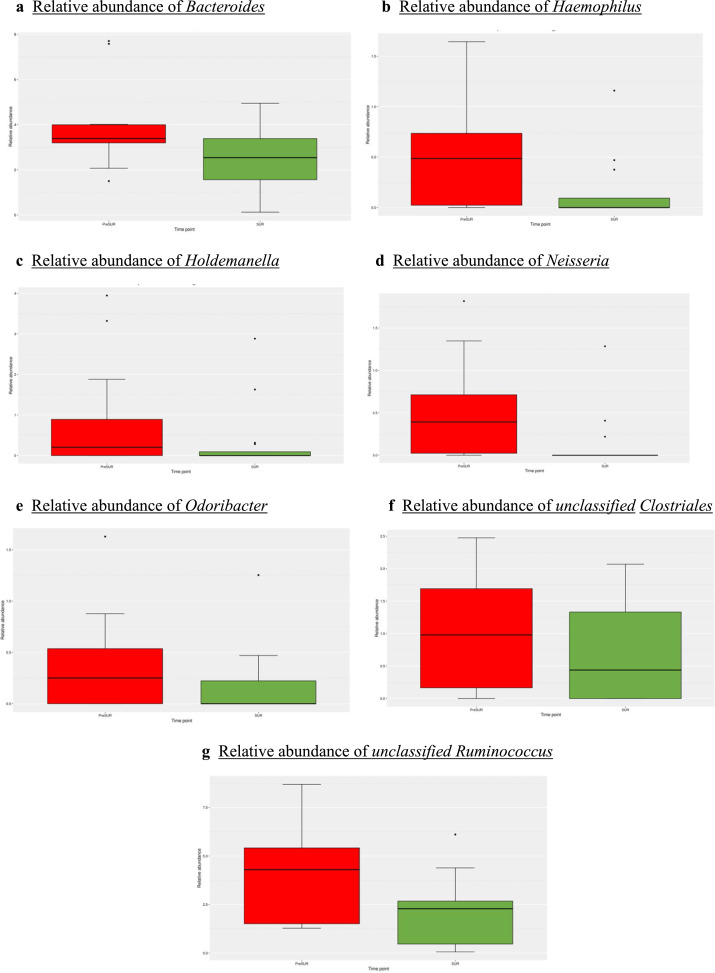
Boxplots of relative abundances PRESURG/SURG (a-g) Boxplots showing relative abundance of Bacteroides, Haemophilus, Holdemanella, Neisseria, Odoribacter, unclassified Clostriales and unclassified Ruminococcus at the time-points before (PreSURG) and during surgery (SUR) in mucosal samples.

Significant decreases were shown for *Bacteroides, Haemophilus, Holdemanella, Neisseria, Odoribacter, unclassified Clostridiales* and *unclassified Ruminococcacaeae* regarding the time points PreSURG (before preparation) and SURG (after MBP/OA+ivAB) (see Fig. 2). The effect size (Cohens's d) was medium to large for *Haemophilus, Bacteroides* and *unclassified Ruminococcacaeae* (see Table 2).

**Table 2:** Significant changes of abundant genera.

Genus	Significance p	Effect size Cohen’s d
Bacteroides	0.01	0.864
Haemophilus	0.047	0.608
Holdemanella	0.004	0.571
Neisseria	0.004	0.584
Odoribacter	0.027	0.537
Unclassified Clostridiales	0.008	0.572
Unclassified Ruminococcacaeae	0.009	0.909

Significance level and effect size (Cohen’s d) of the mainly reduced genera after oral antibiotics in combination with mechanical bowel preparation.

## Discussion

To our knowledge, our study is the only one which investigates the specific effects of MBP/OA+ivAB on the microbiome of patients with colorectal cancer. Similar approaches in healthy patients were investigated by the groups of Heinsen et al. [23] and Nalluri-Butz et al. [24], but the gut microbiome of patients with CRC differs from that of healthy volunteers [25].

Ohigashi et al. [26] showed that the gut microbiome of patients with CRC changes with surgical intervention. Using longitudinal stool samples, they detected a reduction in obligate anaerobes in favour of *Enterobacteriaceae, Enterococcus, Staphylococcus* and *Pseudomonas. Enterococcus faecalis* appears to play a particularly important role in the development of AL. Shogan et al. [13] demonstrated the presence of *Enterococcus faecalis* associated with increased matrix-destructive activity in anastomotic tissue and Lee et al. [17] showed in their retrospective study that *Enterococcus faecalis* is the most common species isolated from the bloodstream or ascites in AL patients after colorectal cancer surgery. One possible reason is that most standard antibiotics (including ertapenem) are less effective against *Enterococc*i. Resistance to aminoglycosides such as parmomomycin was also recently identified using whole genome sequencing [28].

However, further evaluation of our studied patient collective regarding the development of anastomotic insufficiency has shown that *Entercocci* was not significantly increased in the AL samples compared to the patients with sufficient anastomosis [27]. Praagh et al. [14,15] came to a similar conclusion in their study comparing the microbiome of surgical samples from patients with CRC prepared only with MPB. An increased risk for developing an AL was associated with *Bacteroidaceae* (*Bacteroides*) and *Lachnospiracea* (*Blautia, Ruminococcus, Coproccus, Roseburia*) being highly abundant at the time of surgery [14,15]. Regarding *Bacteroides* (*p* = 0.028) and *Ruminococcus* (*p* = 0.029), these results fit very well with our observations (*Bacteroides: p* = 0.01; *unclassified Ruminococcus: p* = 0.009; both with high effect size). However, unlike Praagh et al., in our cohort we found *Prevotella* (*p* < 0.0001) with greater abundance in the AL sample [27]. It is possible that OA treatment can contribute to the selection of anaerobic bacteria which become resistant to metronidazole [29].

In recent years, research has focused on bacteria that can form SCFAs from dietary fibre as supporters for anastomotic healing [30,31]. SCFAs are known as immunoregulatory and energy-providing metabolites in the gastrointestinal tract. Butyrate- in particular- has been shown to have positive effects on anastomotic healing in the colon by improving epithelial proliferation and maintaining barrier function. Most butyrate producers belong to the phylum Firmicutes, in particular *Faecalibacterium, Roseburia, Eubacterium, Anaerostipes, Coprococcus, Subdoligranulum* and *Anaerobutyricum* [30]. With regard to these bacteria, we did not see any significant reduction as a result of the antibiotic treatment, which could possibly be a protective influence. A significant reduction of typical bacteria causing SSI in abdominal surgery such as gram-positive cocci (*Staphylococcus aureus, Streptococci species, Enterococcus species*) and gram-negative *Enterobacteriaceae* could not be detected in our study [32].

An important limitation of our study is the low number of patients. This means that differences in the microbiome due to concomitant diseases such as obesity [33] or diabetes mellitus type II [34] cannot be adequately addressed. The ACS (American College of Surgery) Surgical Risk Score includes patients’ comorbidities and demographic details; using this score, 5 patients were found to have an increased risk of AL and 4 patients an increased risk of SSI in our cohort. Furthermore, no statements can be made about changes in the microbial composition due to (partial) tumour obstruction or the tumour stage. In addition, the significance of the correlation with postoperative complications is limited due to the small patient cohort, even though it has been shown that the patient samples clustered very well preoperatively [27].

An advantage of our study is that we used mucosal samples which have been shown to represent a greater microbial biodiversity, are more reproducible and have greater homogeneity [35,36]. We acknowledge that other microbial sequencing methods, such as metagenomic sequencing, may be better suited to analysing the types of bacterial strains, but there are technical limitations related to the sample material because most of the extracted DNA belongs to humans, not bacteria. Based on our previous experience [27], we have overcome this problem by using 16S rRNA gene amplification which has both advantages and limitations.

## Conclusions

By determining the effects of MBP/OA+ivAB on the microbiome, our study aims to close a gap in research. We were able to demonstrate that MBP/OA+ivAB causes a significant decrease in certain bacteria such as *Bacteroides* and *Ruminococcus* which have been linked to AL in literature. Our work is a pilot study and further studies will be needed to provide in depth view. Identifying the best antibiotic protocol should also be the goal of further evaluation of perioperative microbial drift.

## Ethical approval

All procedures performed in studies involving human participants were in accordance with the ethical standards of the institutional and/or national research committee and with the 1964 Helsinki Declaration and its later amendments or comparable ethical standards. The study was approved by the Bioethics Committee of the Medical University of Leipzig (number 253/19-ek).

## Informed consent

The privacy rights of human subjects have been observed and informed consent was obtained from all individual participants included in the study.

## CRediT authorship contribution statement

**Undine Gabriele Lange:** Writing – review & editing, Writing – original draft, Methodology, Data curation, Conceptualization. **Konrad Lehr:** Writing – review & editing, Software, Methodology, Formal analysis. **René Thieme:** Writing – review & editing. **Albrecht Hoffmeister:** Writing – review & editing, Data curation. **Jürgen Feisthammel:** Writing – review & editing, Data curation. **Ines Gockel:** Writing – review & editing, Conceptualization. **Alexander Link:** Writing – review & editing, Validation, Supervision, Software, Methodology, Formal analysis, Conceptualization. **Boris Jansen-Winkeln:** Writing – review & editing, Validation, Supervision, Data curation, Conceptualization.

## Declaration of competing interest

The authors declare the following financial interests/personal relationships which may be considered as potential competing interests: Alexander Link reports financial support was provided by German Federal Ministry of Education and Research (BMBF). Alexander Link reports was provided by European Regional Development Fund. Alexander Link reports financial support was provided by Janssen Pharmaceuticals Inc. Alexander Link reports financial support was provided by Ferring Pharmaceuticals Inc. Alexander Link reports financial support was provided by LUVO Medical Technologies Inc. The other authors declare that they have no known competing financial interests or personal relationships that could have appeared to influence the work reported in this paper.

## Appendix Supplementary materials

Image, application 1
